# *Rubia tinctorum* root extracts: chemical profile and management of type II diabetes mellitus

**DOI:** 10.1039/d0ra03442h

**Published:** 2020-06-24

**Authors:** Enas E. Eltamany, Mohamed S. Nafie, Dina M. Khodeer, Aya H. H. El-Tanahy, Maged S. Abdel-Kader, Jihan M. Badr, Reda F. A. Abdelhameed

**Affiliations:** Department of Pharmacognosy, Faculty of Pharmacy, Suez Canal University Ismailia 41522 Egypt; Department of Chemistry, Faculty of Science, Suez Canal University Ismailia 41522 Egypt; Department of Pharmacology & Toxicology, Faculty of Pharmacy, Suez Canal University Ismailia 41522 Egypt; Damietta Health Affairs Directorate Damietta Egypt; Department of Pharmacognosy, College of Pharmacy, Prince Sattam Bin Abdulaziz University 173, AlKharj 11942 Saudi Arabia mpharm101@hotmail.com +966545539145; Department of Pharmacognosy, Faculty of Pharmacy, Alexandria University Alexandria 21215 Egypt

## Abstract

The chemical and biological profiling of the root extracts of *Rubia tinctorum* was performed. The activities of different extracts were determined considering the antidiabetic effect against type II diabetes mellitus together with anti-obesity and hepatoprotective effects and lipid profile. The methanolic extract of *Rubia tinctorum* exhibited significant results in decreasing body weight, improving lipid profile, normalizing hyperglycaemia, insulin resistance, hyperinsulinemia. Additionally, it showed enhancement of liver tissue structure and function. The methanolic extract, being the most significant one, was subjected to LC-HRMS analysis to determine its chemical constituents. Finally, the chemical constituents were evaluated by molecular docking study that was carried out to identify the interaction of a panel of 45 compounds *in silico* and to correlate the structures to their anti-diabetic activity. Among the tested compounds, 1-hydroxy-2-hydroxymethyl anthra-quinone and naringenin-7-*O*-glucoside showed the most potent activity as α-amylase inhibitors.

## Abstract 2

The chemical and biological profiling of the root extracts of *Rubia tinctorum* was performed.

## Graphical abstract

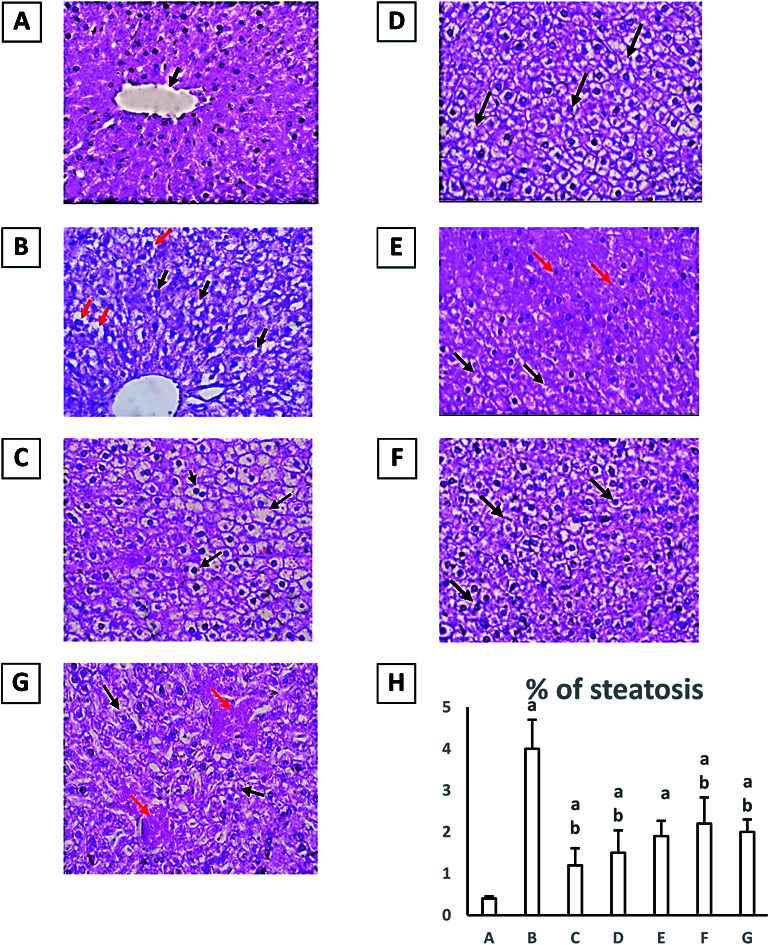

## 1. Introduction

Diabetes mellitus and obesity are important health problems worldwide. They contribute to the development of various pathological processes such as hypertension, cardiovascular diseases, hyperlipidaemia, certain types of cancer and even Alzheimer's.^1,2^ There is a strong correlation between diabetes and obesity, where adipose tissue has an important role in diabetes, a disease characterized by hyperglycemia, insulin hyposecretion, and insulin resistance.^3,4^ The exploitation of phyto-medicine as a therapy for diabetes as well as obesity is a crucial issue for the development of safer alternatives to pharmaceuticals which not only decrease blood glucose but also enhance the antioxidant system.^5^ The genus *Rubia* belongs to the family Rubiaceae and comprises about 70 species.^6^ Previous research on *Rubia* species yielded the isolation and chemical identification of about 250 compounds with different chemical classes which possess diverse pharmacological activities including anti-bacteria, antithrombic, anticancer, anti-inflammatory and anti-oxidant.^7–10^

*Rubia tinctorum* L. (Madder root) was used as a source of red dyes in ancient Egypt.^11^ In addition, several ethnobotanical surveys have reported its use for treatment of various ailments, such as cardiovascular disease,^12,13^ liver pain, diarrhea,^14,15^ rheumatism^16^ and kidney stones.^17,18^ Moreover numerous biological studies have been extensively conducted on *R. tinctorum* and proved its therapeutic potential as antiplatelet aggregation,^19^ antitumor,^20^ hepatoprotective,^21^ vasoconstriction and protective effect on aorta.^22^ The current work was designed to evaluate the antiobesity, antidiabetic and hepatoprotective efficacy of different extracts of *R. tinctorum* along with profiling of potent bioactive components responsible for the antidiabetic activity. In our strategy, we used LC-HRMS technique to identify the phytochemicals in the root extract of *R. tinctorum* then to determine the binding mode of the tested compounds with 1HX0 as α-amylase inhibitor by using molecular docking tool.

## 2. Materials and methods

### 2.1. *In vitro* determination of hypoglycemic activity, liver enzymes and lipid profile

#### 2.1.1. Plant material

The roots of *Rubia tinctorum* were obtained from the Egyptian market and the identity was confirmed in the Faculty of Science, Suez Canal University. A specimen was deposited at Pharmacognosy Department, Faculty of Pharmacy, Suez Canal University, with a code number 2019-RT. Two kilograms of the roots were dried, powdered and extracted with methanol. The extract was dried under vacuum using rotary evaporator to give 250 g of brownish-red methanolic extract (RM). A weight of 160 g of the methanol extract were withdrawn and suspended in 200 mL of distilled water then fractionated with 3 L of each of hexane, chloroform and ethyl acetate successively. Each of the three extracts was dried under vacuum using rotary evaporator to yield 30, 60 and 40 g of hexane (RH), chloroform (RC) and ethyl acetate (RE) extracts respectively.

#### 2.1.2. Experimental animals

Thirty-five male Wistar rats were used in the current study. The base line body weight was in the range of 118–152 g. They were kept in clean cages with temperature equals 21 ± 6 °C and normal light–dark cycle. They had free permission to water and regular diet or HFD. Study protocol was approved by the Committee of Research Ethics at Faculty of Pharmacy in Suez Canal University (license number 202004R2).

#### 2.1.3. Experimental design

Wistar male rats were distributed into seven groups; with 5 rats in every group. First group of rats was assigned as the normal group and received normal chow diet during the experimentation period. Diabetes mellitus type II was induced in the other six groups by modified high fat diet model.^23–25^ The other five groups were fed with HFD (87.7% standard diet, 10% lard fat, 30% glucose) for 7 weeks followed by small dose of streptozotocin (STZ 1% g L^−1^ acetic acid; 30 mg kg^−1^, S. C). After further five days, blood glucose levels are determined to insure the incidence of diabetes mellitus. After that, rats in Group II (diabetic control group) were given distilled water (1 mL per kg per day, p. o.) till the end of experiment. Rats in group III were given pioglitazone (10 mg per kg per day, p. o.) while rats in group IV, V, VI and VII were given the following extracts RM (200 mg kg^−1^), RH (200 mg kg^−1^), RC (200 mg kg^−1^) and RE (200 mg kg^−1^), respectively for further four weeks. After completion of the treatment regimens, final body weight was recorded. The change in body weight was calculated from the following equation: Δbody weight = (the final body weight − the initial body weight)/initial body weight × 100. Similarly, obesity index was calculated by: obesity index = weight of total adipose tissue/final body weight × 100.

#### 2.1.4. Fasting blood glucose determination

Rats were subjected to overnight fasting. Blood specimens were gathered from each rat's tail tip, and fasting glucose was recorded by the use of an automated blood glucometer (Super Glucocard, Japan).

#### 2.1.5. Liver processing

The rats were sacrificed under anaesthesia. Each rat's liver was quickly dissected and washed out of blood with cold saline solution. The weight of livers was measured and the following formula was used for determining the liver index: (liver weight/body weight × 100).

Portion of liver tissue was removed from the biggest hepatic lobe, fixed in formaldehyde and finally stained with hematoxylin and eosin (H&E).

#### 2.1.6. Measurement of serum biochemical parameters

##### 2.1.6.1. Liver enzymes

Spectrophotometrically method was done with marketable kits (Biocon Diagnostic, Germany) to evaluate serum activity of liver enzymes; alanine transaminase enzyme (ALT) (EC 2.6.1.2), aspartate transaminase enzyme (AST) (EC 2.6.1) in accordance with the protocol reported by the manufacturer.^26^

##### 2.1.6.2. Lipid profile

A spectrophotometric assay kits (Spinreact, Spain) were used to measure serum total cholesterol (TC)(CHOD-POD), triglycerides (TGs)(GPO-POD, Líquido), high-density lipoprotein (HDL) (HDLc-P) and low-density lipoprotein (LDL)(LDLc-D) according to the manufacturer's protocol.^27,28^

##### 2.1.6.3. Insulin & leptin ELISA kits

The level of serum insulin and leptin were determined by a rat insulin and leptin ELISA kits (PELOBIOTECH GmbH-Am Klopferspitz 19-82152 planning-Germany) following the manufacturer's protocol. Insulin resistance was determined using the homeostasis model assessment index for insulin resistance (HOMA-IR) index.^29^

#### 2.1.7. Statistical analysis for the data

Results obtained from the current study were expressed as mean ± S. E. M and analysed with the version 16 of SPSS program. A one-way analysis of variance (ANOVA) was used to analyse quantitative variables, followed by the multiple comparison test of Bonferroni. Significant variations were measured at *p* ≤ 0.05.

### 2.2. Preparation of the sample and LC-HRMS analysis

The mobile phase working solution (MP-WS) was prepared from DI-water : methanol : acetonitrile (50 : 25 : 25). One mL of MP-WS was added to 50 mg weighted dry methanolic extract, vortex for 2 min. This step was followed by ultra-sonication for 10 min then centrifugation for 10 min at 10 000 rpm. 20 μL stock (50/1000 μL) was diluted with 1000 μL reconstitution solvent. Finally, the injected concentration was 1 μg μL^−1^ where 10 μLs were injected on positive mode. Also, 10 μL MP-WS were injected as a blank sample. The used mobile phase consisted of (A): 5 mM ammonium formate buffer pH 3 containing 1% methanol and (B): 100% acetonitrile. The flow rate was 0.3 mL min^−1^. The used pre-column was in-line filter disks (Phenomenex, 0.5 μm × 3.0 mm) and the column was X select HSS T3 (Waters, 2.5 μm, 2.1 × 150 mm). Data processing was *via* MS-DIAL3.52. Master view was used for feature (peaks) extraction from total ion chromatogram based on the following criteria: features should have signal-to-noise greater than 5 (non-targeted analysis) and features intensities of the sample-to-blank should be greater than 5.

### 2.3. Molecular docking

Molecular modelling study was carried out to study the interaction of a panel of 28 anthraquinone and 17 flavonoids *in silico* and to correlate the structures to their anti-diabetic activity. Molecular docking study was conducted on a computational software basis using the Molecular Operating Environment (MOE 2014.09 Chemical Computing Group, Canada). The three-dimensional structures of 1HX0 completed with AC1 as alpha-amylase inhibitor was freely accessible from the protein data bank (https://www.rcsb.org/structure/1HX0).^30^ Compounds were chemically optimized and energetically minimized, while the receptor was prepared and manipulated using routine protocol according to Nafie *et al.*^31^ The active sites were defined using grid boxes of appropriate sizes around the co-crystallized ligands. These compounds were docked into the receptor active site, each ligand-receptor complex was tested for binding energy using MOE and interaction analysis using Chimera as a visualizing software.

## 3. Results and discussion

### 3.1. Effect of different *R. tinctorum* extracts and pioglitazone (10 mg kg^−1^) on percent change in body weight and obesity index on type II diabetic rats

Treatment with high fat diet followed with STZ (30 mg kg^−1^) in diabetic group resulted in a significant increase in final body weight (352 ± 7.5), % change in body weight (152 ± 14.1) and obesity index (5.9 ± 0.5) *versus* normal group (210 ± 10) (42.5 ± 9) and (0.83 ± 0.07) respectively at *p* ≤ 0.05 (Table 1). Treatment with pioglitazone (10 mg kg^−1^) for four weeks after induction of diabetes induced significantly decrease in final body weight, % change in body weight and obesity index when compared with diabetic group at *p* ≤ 0.05. Pioglitazone is one member from thiazolidines, as it is PAPR-gamma agonist in particular is known to favorably influence the majority of the components of insulin resistance characteristic of type 2 diabetes mellitus including adiposity, dyslipidaemia, hyperglycaemia and changes in liver and ovaries.^32^ However, its effect in weight gain was previously discussed.^33,34^ On the other hand, its role in decreasing weight gain, enhancing lipid profile and stimulation of lipid mobilization from visceral part to the lower part of body was also reported.^35,36^

**Table 1:** Effect of different *R. tinctorum* extracts and pioglitazone (10 mg kg^−1^) on percent change in body weight and obesity index in the experimental groups of type II diabetic rats^a^

Group	Base line body weight (g)	Final body weight (g)	% change in body weight	Obesity index
Normal	147.5 ± 2.5	210 ± 10	42.5 ± 9	0.83 ± 0.07
Diabetic	140 ± 5	352 ± 7.5^a^	152 ± 14.1^a^	5.9 ± 0.5^a^
Diabetic + pioglitazone (10 mg kg^−1^)	143.5 ± 1.5	227.5 ± 17.6^b^	58 ± 13.6^b^	2 ± 0.36^b^
Diabetic + RM (200 mg kg^−1^)	167.5 ± 7.5	308 ± 8^a,b,c^	84 ± 3.4^a,b^	2.5 ± 0.89^a,b^
Diabetic + RH (200 mg kg^−1^)	140 ± 2	333.5 ± 16.6^a,c,e^	138 ± 8.2^a,c,d^	5.1 ± 0.17^a,c,d^
Diabetic + RC (200 mg kg^−1^)	141 ± 4	291.5 ± 18.6^a–c^	106 ± 7.1^a–c^	3.7 ± 0.1^a–c^
Diabetic + RE (200 mg kg^−1^)	145 ± 10	297.3 ± 3^a–c^	106 ± 15.9^a–c^	5.3 ± 0.03^a,c–e^

a Results are expressed as mean ± S. E. M. and analysed using one-way ANOVA followed by Bonferroni's test for multiple comparisons. ^a^*P* ≤ 0.05 *versus* normal group. ^b^*P* ≤ 0.05 *versus* diabetic group. ^c^*P* ≤ 0.05 *versus* diabetic + pioglitazone (10 mg kg^−1^) group. ^d^*P* ≤ 0.05 diabetic + RM (200 mg kg^−1^) group, ^e^*P* ≤ 0.05 diabetic + RH (200 mg kg^−1^) group. *n* = 5.

The current results are in agreement with these articles, as pioglitazone treatment reduced total body weight and decreased liver-fat resulting in elevation of insulin sensitivity in these tissues. Additionally, the effect of pioglitazone is related to the correct choice of its dose as lower and higher doses of pioglitazone may exert no or adverse action like sodium water retention and weight gain.^37^ So, the dose of pioglitazone should be monitored and well selected.^23^ In the current study the selected dose was (10 mg kg^−1^) which is considered to be medium dose and has significant effect in all measured parameters.

Similarly, diabetic rats treated with RM, RC or RE (each 200 mg kg^−1^) extracts showed a significant decrease in final body weight and % change in body weight, however, only diabetic rats treated with RM (200 mg kg^−1^) and RC (200 mg kg^−1^) showed a significant improvement in obesity index compared with diabetic group. On the other hand, the group treated with the extract RH (200 mg kg^−1^) couldn't show any significant enhancement in final body weight, % change in body weight or obesity index in comparison with diabetic group at *p* ≤ 0.05. Moreover, the results achieved by the treatment with the extract RM (200 mg kg^−1^) were the best in improving the decrease % change in body weight and obesity index (Table 1).

### 3.2. Effect of different *R. tinctorum* extracts and pioglitazone (10 mg kg^−1^) on blood glucose level, serum insulin, insulin resistance and serum leptin level on type II diabetic rats

The current results showed significant increases in blood glucose level (mM L^−1^), serum insulin level (ng L^−1^), HOMA-IR and serum leptin level (ng L^−1^) in diabetic group in comparison with normal group at *p* ≤ 0.05 (Table 2). However, treatment with pioglitazone (10 mg kg^−1^) significantly decreased blood glucose level (mM L^−1^), serum insulin level (ng L^−1^), HOMA-IR and serum leptin level (ng L^−1^) when compared to diabetic group at *p* ≤ 0.05. Additionally, treatment with RM, RH, RC and RE (each of 200 mg kg^−1^) significantly induced a decrease in blood glucose level (mM L^−1^), serum insulin level (ng L^−1^), HOMA-IR and serum leptin level (ng L^−1^) when compared to diabetic group at *p* ≤ 0.05. However, the most significant results were obtained from the group treated with RM (200 mg kg^−1^) concerning normalization of the level of serum leptin (ng L^−1^) at *p* ≤ 0.05 (Table 2).

**Table 2:** Effect of different *R. tinctorum* extracts and pioglitazone (10 mg kg^−1^) on blood glucose level (mM L^−1^), serum insulin (ng L^−1^), homeostatic model assessment of insulin resistance (HOMA-IR) and serum leptin level (ng L^−1^) in the experimental groups of type II diabetic rats^a^

Group	Blood glucose level (mM L^−1^)	Serum insulin (ng L^−1^)	HOMA-IR	Serum leptin (ng L^−1^)
Normal	97 ± 3.9	2.4 ± 0.05	14.3 ± 0.87	3.8 ± 0.3
Diabetic	172 ± 24^a^	7.6 ± 0.29^a^	74.79 ± 16.7^a^	14 ± 1^a^
Diabetic + pioglitazone (10 mg kg^−1^)	110 ± 5.6^b^	3.9 ± 0.02^a,b^	26.5 ± 1.5^b^	4.9 ± 0.3^b^
Diabetic + RM (200 mg kg^−1^)	99 ± 2^b^	4 ± 0.06^a,b^	29.8 ± 0.99^b^	5.6 ± 0.1^b^
Diabetic + RH (200 mg kg^−1^)	125 ± 7.6^b^	4.1 ± 0.1^a,b^	34.6 ± 2.8^b^	10.5 ± 0.5^a–d^
Diabetic + RC (200 mg kg^−1^)	111 ± 2.0^b^	4.2 ± 0.16^a,b^	30.14 ± 1.6^b^	8.8 ± 0.7^a–d^
Diabetic + RE (200 mg kg^−1^)	102 ± 0.66^b^	4 ± 0.6^a,b^	25.5 ± 0.58^b^	8.8 ± 0.15^a–d^

a Results are expressed as mean ± S. E. M. and analysed using one-way ANOVA followed by Bonferroni's test for multiple comparisons. ^a^*P* ≤ 0.05 *versus* normal group. ^b^*P* ≤ 0.05 *versus* diabetic group. ^c^*P* ≤ 0.05 *versus* diabetic + pioglitazone (10 mg kg^−1^) group. ^d^*P* ≤ 0.05 diabetic + RM (200 mg kg^−1^) group. *n* = 5.

### 3.3. Effect of different *R. tinctorum* extracts and pioglitazone (10 mg kg^−1^) on liver index, serum liver enzymes level, hepatic tissue histopathological changes and percent of steatosis on type II diabetic rats

In the current study, diabetic group showed a significant increase in liver index with a value (3.5 ± 0.2) *versus* (2.3 ± 0.1) in normal group at *p* ≤ 0.05 (Table 3). Diabetic group showed elevation in serum liver enzymes AST and ALT in comparison with normal group at *p* ≤ 0.05. Furthermore, treatment of diabetic rats with only pioglitazone (10 mg kg^−1^) or the extract RM (200 mg kg^−1^) for four weeks could significantly normalize the liver index in comparison with diabetic group at *p* ≤ 0.05. Additionally, treatment with pioglitazone (10 mg kg^−1^), RM, RH, RC and RE (each 200 mg kg^−1^) significantly induced a decrease in both two serum liver enzymes ALT and AST in comparison with diabetic group at *p* ≤ 0.05.

**Table 3:** Effect of different *R. tinctorum* extracts and pioglitazone (10 mg kg^−1^) on liver index, serum liver enzymes level alanine transaminase (ALT) and aspartate transaminase (AST) in the experimental groups of type II diabetic rats^a^

Group	Liver index	AST (U L^-1^)	ALT (U L-^1^)
Normal	2.3 ± 0.1	39 ± 1	34.5 ± 1.5
Diabetic	3.5 ± 0.2^a^	92.5 ± 0.5^a^	78 ± 2.02^a^
Diabetic + pioglitazone (10 mg kg^−1^)	2.5 ± 0.3^b^	50.5 ± 1.5^a,b^	26.5 ± 2.5^b^
Diabetic + RM (200 mg kg^−1^)	2.5 ± 0.02^b^	35 ± 1.01^b,c^	30 ± 1.01^b^
Diabetic + RH (200 mg kg^−1^)	3.9 ± 0.07^a,c,d^	72 ± 1.01^a–d^	55 ± 1.01^a–d^
Diabetic + RC (200 mg kg^−1^)	3.07 ± 0 0.04^a,c–e^	65.5 ± 2.5^a–e^	46.5 ± 1.5^a–e^
Diabetic + RE (200 mg kg^−1^)	3.35 ± 0.02^a,c–e^	66 ± 1.01^a–e^	46 ± 1.01^a–e^

a Results are expressed as mean ± S. E. M. and analyzed using one-way ANOVA followed by Bonferroni's test for multiple comparisons. ^a^*P* ≤ 0.05 *versus* normal group. ^b^*P* ≤ 0.05 *versus* diabetic group. ^c^*P* ≤ 0.05 *versus* diabetic + pioglitazone (10 mg kg^−1^) group. ^d^*P* ≤ 0.05 diabetic + RM (200 mg kg^−1^) group, ^e^*P* ≤ 0.05 diabetic + RH (200 mg kg^−1^) group. *n* = 5.

**Fig. 1:**
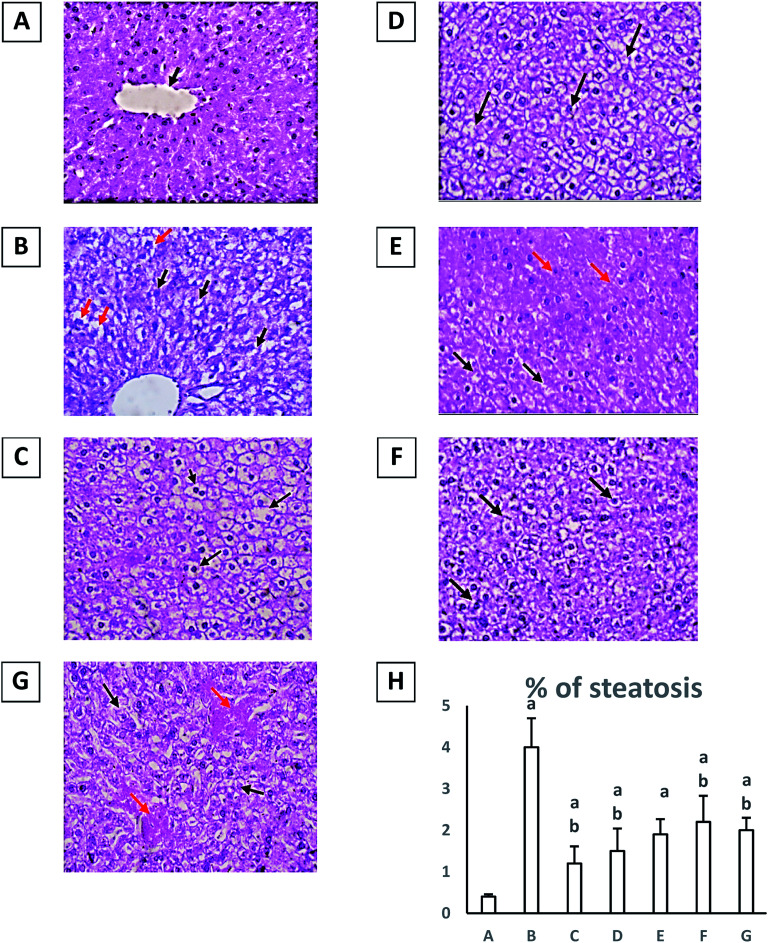
Histopathological picture for hepatic specimens stained with hematoxylin and eosin with magnification power 40×. (A) Histopathology images for liver sections from normal group uniform hepatocytes arranged in plates radiating from central vein (black arrows) (H&E, 40×). (B) Diabetic group in which hepatocytes show evidence of injury; hydropic degeneration (black arrows) and steatosis (red arrows) (H&E, 40×). (C) Diabetic + pioglitazone (10 mg kg^−1^) shows mild histopathological changes (H&E, 40×). (D) Diabetic + RM (200 mg kg^−1^) group which shows mild hitopathological changes. (E) Diabetic + RH (200 mg kg^−1^) group which shows mild hydropic degeneration of hepatocytes (black arrows) and many hepatocytes show uniform morphology (red arrows) (H&E, 40×). (F) Diabetic + RC (200 mg kg^−1^) which shows moderate degeneration in hepatocytes (black arrows) (H&E, 40×). (G) Diabetic + RE (200 mg kg^−1^) which shows moderate hydropic degeneration of hepatocytes (black arrows). Congested sinusoids are seen (red arrows) (H&E, 40×). (H) Effect of different extracts of *Rubia tinctorum* and pioglitazone (10 mg kg^−1^) on percent of liver steotosis. (A) Normal group. (B) Diabetic group. (C) Diabetic + pioglitazone (10 mg kg^−1^). (D) Diabetic + RM (200 mg kg^−1^). (E) Diabetic + RH (200 mg kg^−1^). (F) Diabetic + RC (200 mg kg^−1^). (G) Diabetic + RE (200 mg kg^−1^). Results are expressed as mean ± S. E. M. and analyzed using one-way ANOVA followed by Bonferroni's test for multiple comparisons. ^a^*P* ≤ 0.05 *versus* normal group. ^b^*P* ≤ 0.05 *versus* diabetic group. *n* = 5.

Finally, diabetic group showed evidence of injury; hydropic degeneration (black arrows) and a significant increase in percent of steatosis (red arrows) (H&E, 40×) when compared to normal group at *p* ≤ 0.05 (Fig. 1). Treatment with either pioglitazone (10 mg kg^−1^) or any of the *Rubia* extracts showed enhancement in liver architecture and a significant decrease in percent of steatosis with a significant reduction in hydropic degeneration of hepatocytes (black arrows) and many hepatocytes show uniform morphology (red arrows) (H&E, 40×) in comparison with diabetic group at *p* ≤ 0.05 (Fig. 1). However, the results obtained by treatment with the extract RM were the best and closer to the normal group (Table 3).

### 3.4. Effect of different *R. tinctorum* extracts and pioglitazone (10 mg kg^−1^) on lipid profile, serum triglycerides (TG), total cholesterol (TC), high-density lipoprotein (HDL) and low-density lipoprotein (LDL) on type II diabetic rats

Treatment with high fat diet followed with STZ (30 mg kg^−1^) in diabetic group resulted in a significant increase in serum triglycerides (TG) (mg dL^−1^), total cholesterol (TC) (mg dL^−1^) and low-density lipoprotein (LDL) (mg dL^−1^) and a significant decrease in high-density lipoprotein (HDL) (mg dL^−1^) in comparison with normal group at *p* ≤ 0.05 (Table 4). Treatment with pioglitazone (10 mg kg^−1^) resulted in a significant decrease in serum triglycerides (TG) (mg dL^−1^), total cholesterol (TC) (mg dL^−1^) and low-density lipoprotein (LDL) (mg dL^−1^) and a significant increase in high-density lipoprotein (HDL) (mg dL^−1^) in comparison with diabetic group at *p* ≤ 0.05.

**Table 4:** Effect of different *R. tinctorum* extracts and pioglitazone (10 mg kg^−1^) on lipid profile, serum triglycerides (TG), total cholesterol (TC), high-density lipoprotein (HDL) and low-density lipoprotein (LDL) in the experimental groups of type II diabetes in rats^a^

Group	Serum TG (mg dL^−1^)	Serum TC (mg dL^−1^)	HDL (mg dL^−1^)	LDL (mg dL^−1^)
Normal	54.5 ± 3.5	63 ± 3.03	45 ± 11	24.5 ± 0.5
Diabetic	161 ± 16.1^a^	116.5 ± 3.5^a^	20 ± 2.02^a^	55.5 ± 4.5^a^
Diabetic + pioglitazone (10 mg kg^−1^)	42.5 ± 2.5^b^	88.5 ± 1.6^a,b^	40.5 ± 3.5^b^	30 ± 2.02^b^
Diabetic + RM (200 mg kg^−1^)	68 ± 1.01^b,c^	72 ± 1.01^a–c^	36 ± 0.5^b^	23 ± 1.01^b^
Diabetic + RH (200 mg kg^−1^)	98.5 ± 0.5^a–d^	89.5 ± 0.5^a,b,d^	30.5 ± 0.5^a^	46.5 ± 0.5^a–d^
Diabetic + RC (200 mg kg^−1^)	85 ± 2.02^a–c^	82.5 ± 0.5^a–e^	26 ± 1.01^a,c^	45.5 ± 3.5^a–d^
Diabetic + RE (200 mg kg^−1^)	80.5 ± 0.5^a–c^	76.5 ± 0.5^a–c,e^	26.5 ± 0.5^a^	39.5 ± 0.5^a–d^

a Results are expressed as mean ± S. E. M. and analyzed using one-way ANOVA followed by Bonferroni's test for multiple comparisons. ^a^*P* ≤ 0.05 *versus* normal group. ^b^*P* ≤ 0.05 *versus* diabetic group. ^c^*P* ≤ 0.05 *versus* diabetic + pioglitazone (10 mg kg^−1^) group. ^d^*P* ≤ 0.05 diabetic + RM (200 mg kg^−1^) group, ^e^*P* ≤ 0.05 diabetic + RH (200 mg kg^−1^) group. *n* = 5.

Furthermore, all extracts induced a significant decrease in serum triglycerides (TG) (mg dL^−1^), total cholesterol (TC) (mg dL^−1^) and low-density lipoprotein (LDL) (mg dL^−1^) at *p* ≤ 0.05 (Table 4).

Moreover, treatment with RM (200 mg kg^−1^) resulted in a significant improvement and normalization in high-density lipoprotein (HDL) serum level (mg dL^−1^) in comparison with diabetic group at *p* ≤ 0.05 (Table 4).

Accordingly, the results obtained from treatment of the diabetic rats with the methanolic extract of *Rubia tinctorum* were the best and more close to normal group and pioglitazone treated group either in decreasing body weight, obesity, improving lipid profile, normalization hyperglycaemia, insulin resistance, hyperinsulinemia or in enhancing liver tissue structure and function.

### 3.5. LC-HRMS analysis

**Fig. 2:**
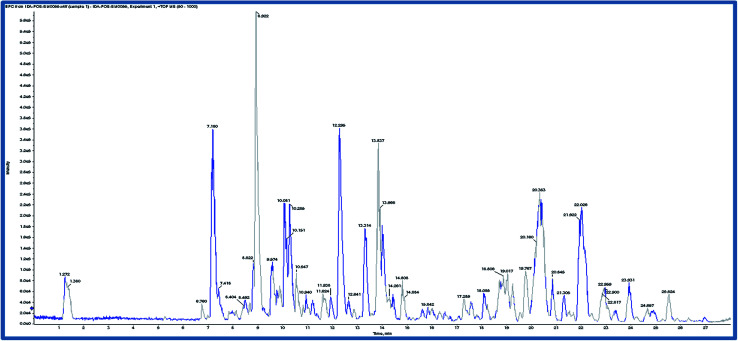
Chromatogram of methanolic root extract of *Rubia tinctorum* in positive mode ion.

Based on the results of the biological activities of different extracts of *R. tinctorum* which revealed that the best extract was the methanolic one, accordingly, the methanolic extract was subjected to LC-HRMS analysis to detect its chemical constituents (Fig. 2). The results showed 45 hits as indicated in Tables 5 and 6.

**Table 5:** Anthraquinones previously isolated from *Rubia tinctorum* root and detected in the extract by LC-HRMS analysis (positive mode)^a^

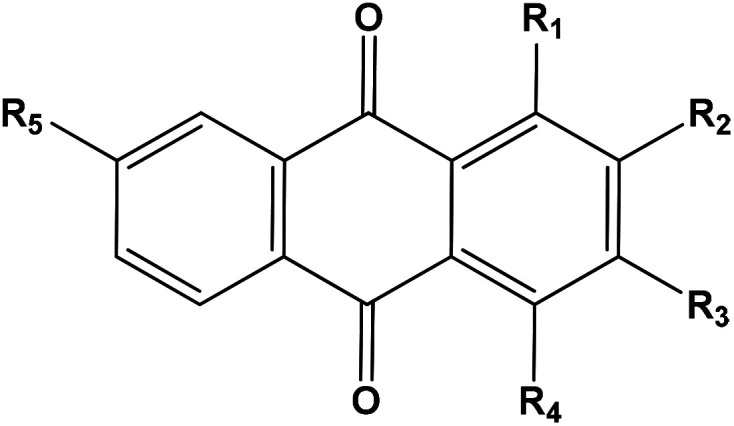								
*R*_*t*_ (min)	Molecular formula	*m*/*z* [*M* + H]^+^	Name of the compound	Substituents				
				R_1_	R_2_	R_3_	R_4_	R_5_
7.18	C_26_H_28_O_14_	565.1557	Lucidin-3-*O*-primveroside	OH	CH_2_OH	*O*-primverose		H
8.82	C_25_H_26_O_13_	535.1451	Ruberythric acid	OH	*O*-Glucose (6-1)xylose	H		H
8.92	C_15_H_8_O_7_	301.0348	Pseudopurpurin	OH	COOH	OH	OH	H
9.57	C_15_H_8_O_6_	285.0399	Munjistin	OH	COOH	OH		H
10.08*	C_14_H_8_O_5_	257.045	Purpurin	OH	OH	H	OH	H
10.28*	C_14_H_8_O_5_	257.045	Anthragallol	OH	OH	OH	H	H
12.29**	C_14_H_8_O_4_	241.0501	Alizarin	OH	OH	H	H	H
12.31**	C_14_H_8_O_4_	241.0501	Xanthopurpurin	OH	H	OH	H	H
12.64	C_15_H_10_O_5_	271.0606	1,4-Dihydroxy-2-hydroxymethyl anthraquinone	OH	CH_2_OH	H	OH	H
12.64	C_14_H_8_O_3_	225.0551	2-Hydroxy anthraquinone	H	OH	H	H	H
13.31	C_15_H_10_O_4_	255.0657	Rubiadin	OH	CH_3_	OH	H	H
13.96	C_17_H_14_O_5_	299.0919	1,4-Dihydroxy-2-ethoxymethyl anthraquinone	OH	CH_2_OC_2_H_5_	H	OH	H
13.77***	C_15_H_10_O_5_	271.0606	Anthragallol-3-methylether	OH	OH	OCH_3_	H	H
13.83***	C_15_H_10_O_5_	271.0606	Lucidin	OH	CH_2_OH	OH	H	H
18.08****	C_15_H_10_O_3_	239.0708	1-Hydroxy-2-methyl AQ	OH	CH_3_	H	H	H
18.75****	C_15_H_10_O_3_	239.0708	7-Hydroxy-2-methyl AQ	H	CH_3_	H	H	OH
18.80^#^	C_15_H_10_O_4_	255.0657	Xanthopurpurin-3-methylether	OH	H	OCH_3_	H	H
19.02^#^	C_15_H_10_O_4_	255.0657	Alizarin-1-methyl ether	OCH_3_	OH	H	H	H
19.53^#^	C_15_H_10_O_4_	255.0657	Alizarin -2-methyl ether	OH	OCH_3_	H	H	H
19.77^#^	C_15_H_10_O_4_	255.0657	Xanthopurpurin-1-methylether	OCH_3_	H	OH	H	H
20.16	C_15_H_10_O_4_	255.0657	1-Hydroxy-2-hydroxymethyl anthraquinone	OH	CH_2_OH	H	H	H
20.5	C_16_H_12_O_5_	285.0763	Anthragallol-2,3-dimethylether	OH	OCH_3_	OCH_3_	H	H
20.84	C_15_H_10_O_3_	239.0708	2-Methoxy- anthraquinone	H	OCH_3_	H	H	H
21.30^##^	C_16_H_12_O_4_	269.0814	Alizarin-dimethyl ether	OCH_3_	OCH_3_	H	H	H
21.40^##^	C_16_H_12_O_4_	269.0814	Xanthopurpurin dimethylether	OCH_3_	H	OCH_3_	H	H
23.9^###^	C_16_H_12_O_3_	253.0864	1-Methoxy methyl anthraquinone	CH_2_OCH_3_	H	H	H	H
24.12^###^	C_16_H_12_O_3_	253.0864	1-Methoxy-2-methyl anthraquinone	OCH_3_	CH_3_	H	H	H
25.52	C_17_H_14_O_3_	266.0943	2-Ethoxymethyl anthraquinone	H	CH_2_OC_2_H_5_	H	H	H

a *, **, ***, ****, ^#^, ^##^, ^###^: interchangeable values.

**Table 6:** Flavonoids of *Rubia tinctorum* root extract identified by LC-HRMS analysis (positive mode)

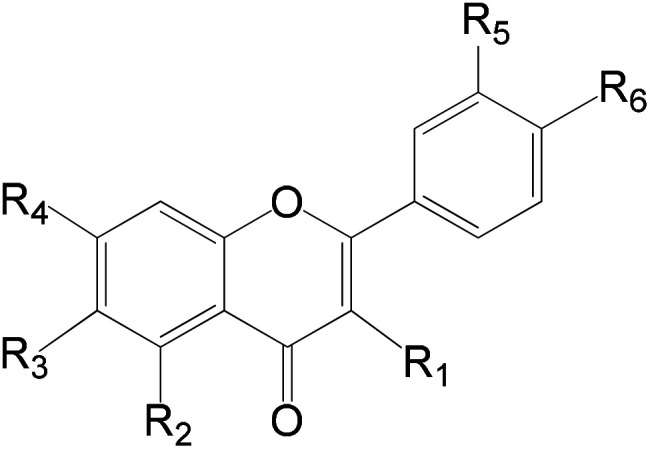									
*R*_*t*_ (min)	Molecular formula	*m*/*z* [*M* + *H*]^+^	Name of the compound	Substituents					
				R_1_	R_2_	R_3_	R_4_	R_5_	R_6_
7.44	C_27_H_30_O_16_	609.1456	Luteolin-3-7-diglucoside	H	OH	H	*O*-Glucose	*O*-Glucose	OH
8.67	C_27_H_30_O_15_	593.1507	Kaempferol-7-neohesperidoside	H	OH	H	*O*-Neohesperidose	OH	OH
9.41	C_28_H_32_O_15_	607.1663	Hesperetin-7-*O*-neohesperidoside	H	OH	H	*O*-Neohesperidose	OH	OCH_3_
10.89	C_28_H_32_O_14_	591.1714	Acacetin 7-rutinoside	H	OH	H	*O*-Rutinose	H	OH
11.30	C_21_H_18_O_11_	445.0771	Baicalein-7-*O*-glucuronide	H	OH	OH	*O*-Glucoronic acid	H	H
11.59	C_21_H_20_O_11_	447.0928	Luteolin-7-*O*-glucoside	H	OH	H	*O*-Glucose	OH	OH
11.60	C_21_H_22_O_10_	433.1135	Naringenin-7-*O*-glucoside	H	OH	H	*O*-Glucose	H	OH
12.30	C_20_H_18_O_11_	433.0771	Quercetin-3-D-xyloside	*O*-Xylose	OH	H	OH	OH	OH
12.36	C_21_H_20_O_11_	447.0928	Quercitrin	*O*-Glucose	OH	H	OH	OH	OH
13.63	C_21_H_20_O_10_	431.0978	Kaempferol-3-*O*-l-rhamnoside	*O*-Rhamnose	OH	H	OH	H	OH
13.77	C_22_H_22_O_12_	477.1033	Isorhamnetin-3-*O*-glucoside	*O*-Glucose	OH	H	OH	OCH_3_	OH
13.83	C_15_H_10_O_6_	285.0399	Luteolin	H	OH	H	OH	OH	OH
14.77	C_15_H_12_O_5_	271.0607	Naringenin	H	OH	H	OH	H	OH
14.90	C_15_H_10_O_5_	269.045	Apigenin	H	OH	H	OH	H	OH
15.84	C_16_H_12_O_6_	299.0556	Hesperetin	H	OH	H	OH	OH	OCH_3_
15.61	C_16_H_12_O_5_	283.0607	Acacetin	H	OH	H	OH	H	OCH_3_
9.82	C_28_H_36_O_15_	611.1976	Neohesperidin dihydrochalcon	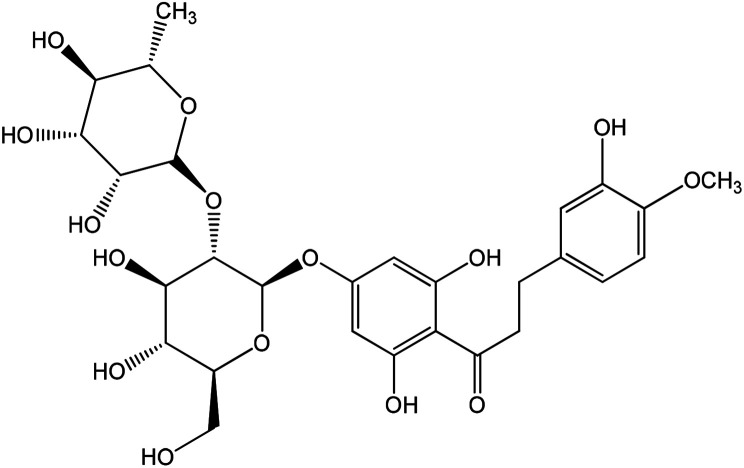					

The anthraquinones were previously reported to be major components of *Rubia* species specially and family Rubiaceae generally.^38^Table 5 revealed the presence of 28 anthraquinones. All of the detected compounds were previously reported to be isolated from the *Rubia tinctorum.*^38–41^

On the other hand, a number of anthraquinones were previously reported to be isolated from the same plant as for example nor damnacanthal and lucidin methyl ether^42^ were not recorded her in the present study. Additionally, family Rubiaceae revealed the presence of a number of flavonoids of which derivatives of quercetin, rhamnetin, isorhamnetin, apigenin and kaempferol are the most common.^42^ In this work a number of 17 flavonoids were recorded as minor constituents of *Rubia tinctorum* (Table 6). On the other hand, rutin which was previously reported to be isolated from *R. tinctorum*^38^ was not detected here, and instead, quercetrin (which could be considered as a secondary glycoside of rutin) was recorded.

### 3.6. Docking study

**Fig. 3:**
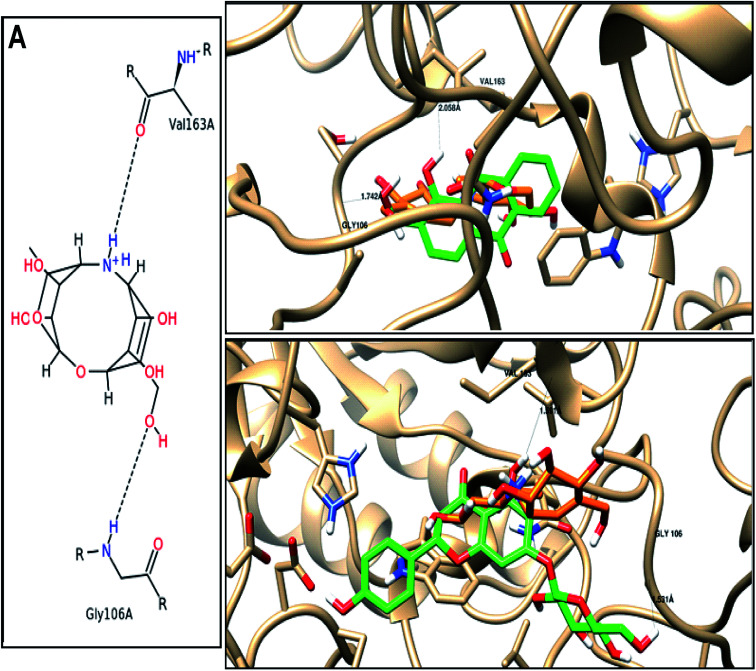
Binding disposition and ligand–receptor interactions of (A) co-crystallized ligand (AC1), and the two high affinity. Docked compounds; (B) 1-hydroxy-2-hydroxymethyl AQ, and (C) naringenin-7-*O*-glucoside inside the 1HX0 binding site as alpha-amylase inhibitor. Co-crystallized ligand (orange), and docked compounds (green).

α-Amylase has been considered as an important therapeutic target for the management of type 2 diabetes mellitus, hence, we aimed to elucidate the binding mode of the tested compounds with 1HX0 as α-amylase inhibitor.^43^ We performed induced fit molecular docking studies with the compounds under investigation. The docking results with docking scores, and the hydrogen bonded residues are given in Table 7. Additionally, 3D representative images of one of the high binding affinities of both anthraquinone and flavonoids compared to AC1 as the co-crystallized ligand are shown in Fig. 3.

**Table 7:** Docking results of the tested compounds with high and mild inside 1HX0 binding site as α-amylase inhibitor compared to AC1 as the co-crystallized ligand. Co-crystallized ligand (AC1) inside the binding site of 1HX0 forms 2 HB with the key amino acids Gly 106 and Val 163^a^

Group	Binding affinity	Identified compound	Binding energy (kcal mol^−1^)	Ligand–receptor interactions with
Anthraquinone*	High	1-Hydroxy-2-hydroxymethyl AQ^#^	−13.92	2 HB with Gly 106 and Val 163
		Ruberythric acid	−21.03	2 HB with Val 163
	Mild	1,4-Dihydroxy-2-ethoxymethyl anthraquinone	−9.05	1 HB with either Val 163 or Gly 106
		2-Hydroxy anthraquinone	−9.36	
		2-Methoxy-anthraquinone	−10.43	
		Lucidin-3-*O*-primveroside	−24.9	
		Lucidin	−11.9	
		Purpurin	−14.78	
		Xanthopurpurin dimethylether	−11.34	
		Xanthopurpurin	−11.68	
		Rubiadin	−15.45	
Flavonoids*	High	Baicalein-7-*O*-glucuronide	−19.66	2 HB with Val 163
		Kaempferol-3-*O*-l-rhamnoside	−20.95	2 HB with Val 163
		Naringenin-7-*O*-glucoside^#^	−16.16	2 HB with Gly 106 and Val 163
		Neohesperidin dihydrochalcon	−23.56	2 HB with Gly 106
	Mild	Acacetin 7-rutinoside	−21.21	1 HB with either Val 163 or Gly 106
		Apigenin	−16.59	
		Hesperetin	−20.98	
		Hesperetin-7-*O*-neohesperidoside	−25.20	
		Isorhamnetin-3-*O*-glucoside	−22.21	
		Kaempferol-7-neohesperidoside	−17.04	
		Luteolin	−18.08	
		Luteolin-3-7-diglucoside	−18.38	
		Luteolin-7-*O*-glucoside	−27.45	
		Naringenin	−17.67	
		Quercetin-3-D-xyloside	−19.95	
		Quercetin	−20.89	

a ^#^Highly-bonded interactive docked compounds in the same way like AC1. *The rest of compounds of both groups weren't able to bind with the receptor pocket.

As shown in Table 7, most of the tested derivative were docked and bound to amino acids of the receptor binding site with high and mild binding affinities (energies), while other derivatives couldn't be docked. In reference to the co-crystallized ligand (AC1) forms two major interactions with the Val 163 and Gly 106 as the key amino acids residues, we found two of the anthraquinones with high binding affinity (−13.92–21.03 kcal mol^−1^), and four flavonoids (−16.16–23.56 kcal mol^−1^) towards α-amylase inhibition by forming the same key interactions. Nine of the anthraquinones, and twelve of flavonoids showed mild binding affinities (−9.05–15.45 kcal mol^−1^) and (−16.59–27.45 kcal mol^−1^), respectively, as they form only one hydrogen bond with either Val 163 or Gly 106.

As shown in Fig. 3, three-dimensional representation of two highly docked compounds as two active leads relative to the AC1 with moieties of ligand and receptor involved in the interaction, interaction-type, bond-length for each docking procedure. Among anthraquinone, 1-hydroxy-2-hydroxy-methyl anthraquinone forms two hydrogen bonds through the hydroxyl groups as H-donor with Val 163 with bonds length 2.05 A, and H-acceptor with Gly 106 with bonds length 1.74 A. Among flavonoids, naringenin-7-*O*-glucoside forms two hydrogen bonds through the hydroxyl groups as H-donors with Val 163 and Gly 106 with bonds length 1.59 and 1.53 Å.

From docking study, we conclude the good affinity of compounds under investigation through their hydroxyl and carbonyl active groups that bind inside the tested target (α-amylase inhibition compared to AC1), which is correlated to anti-diabetic activity.

In addition to α-amylase inhibition activity, most of the compounds detected in the extract proved to possess antioxidant and anti-inflammatory activities.^44–46^ It is well known that both activities play an important role in treatment of diabetes mellitus.^47,48^ This also can justify the activity of the methanolic extract of *Rubia tinctorum* which accumulate a great number of compounds showing antioxidant, anti-inflammatory and α-amylase inhibition activities.

## 4. Conclusions

The current study proved that the methanolic extract of *Rubia tinctorum* showed significant results in decreasing body weight, improving lipid profile, normalizing hyperglycaemia, insulin resistance, hyperinsulinemia in addition to enhancing liver tissue structure and function. There activities could be attributed to its chemical constituents that exert antioxidant, anti-inflammatory and α-amylase inhibition activities. As indicated by the docking study, 1-hydroxy-2-hydroxymethyl anthraquinone and naringenin-7-*O*-glucoside were the most potent as α-amylase inhibitors.

## Conflicts of interest

There are no conflicts to declare.

## Supplementary Material

## References

[cit1] Petrie J. R., Guzik T. J., Touyz R. M. (2018). Can. J. Cardiol..

[cit2] Khalaf S. S., Hafez M. M., Mehanna E. T., Mesbah N. M., Abo-Elmatty D. M. (2019). Int. J. Res. Pharm. Biomed. Sci..

[cit3] Saad B. , Zaid H., Shanak S. and Kadan S., in Anti-diabetes and Anti- obesity Medicinal Plants and Phytochemicals, Springer Nature, Switzerland, 2017, vol. 1, pp. 3–19

[cit4] Shi Y., Hu F. B. (2014). Lancet.

[cit5] Sandborn W. J., Faubion W. A. (2000). Curr. Gastroenterol. Rep..

[cit6] Abdel-Khalik K. N., El-Ghani M. M., Elkordy A. R. (2008). Turk. J. Bot..

[cit7] Tan N. H., Zhou J. (2006). Chem. Rev..

[cit8] Zhao S. M., Kuang B., Fan J. T., Yan H., Xu W. Y., Tan N. H. (2011). Chimia.

[cit9] Xu K., Wang P. L., Yuan B., Cheng Y. T., Li Q., Lei H. M. (2013). Chem. Cent. J..

[cit10] Xu K., Wang P. L., Wang L., Liu C. M., Xu S. X., Cheng Y. T., Wang Y. H., Li Q., Lei H. M. (2014). Chem. Biodiversity.

[cit11] Ahmed H. E., Tahoun I. F., Elkholy I., Shehata A. B., Ziddan Y. (2017). Dyes Pigm..

[cit12] Bellakhdar J., Claisse R., Fleurentin J., Younos C. (1991). J. Ethnopharmacol..

[cit13] Jouad H., Haloui M., Rhiouani H., El Hilaly J., Eddouks M. (2001). J. Ethnopharmacol..

[cit14] Eddouks M., Maghrani M., Lemhadri A., Ouahidi L. M., Jouad H. (2002). J. Ethnopharmacol..

[cit15] El Haouari M., Rosado J. A. (2016). Phytother. Res..

[cit16] Adams M., Berset C., Kessler M., Hamburger M. (2009). J. Ethnopharmacol..

[cit17] Gunther R. T. , Goodyer J., The Greek herbal of Dioscorides*.* New York: Hafner Publishing, 1959

[cit18] Agarwal K., Varma R. (2015). J. Ethnopharmacol..

[cit19] Marhoume F. Z., Laaradia M. A., Younes Z., Laadraoui J., Oufkir S., Aboufatima R., Chait A., Bagri A. (2019). J. Ethnopharmacol..

[cit20] Rashan L., Lukmanul H., Heinz F., Gerhardt K., Irmgard M., Mohammed A., Sidgi H. (2018). Jordan J. Biol. Sci..

[cit21] Marhoume F. Z., Younes Z., Boufous H., Errafiy N., Ait Laaradia M., Laadraoui J., Hakmaoui A., Abdellah B., Abderrahman C. (2017). Eur. J. Med. Plants.

[cit22] Nejat H., Sedaghat K., Vakili A., Jarrahi M., Khorasani M. Z. (2017). Jundishapur J. Nat. Pharm. Prod..

[cit23] Khodeer D. M., Zaitone S. A., Farag N. E., Moustafa Y. M. (2016). Can. J. Physiol. Pharmacol..

[cit24] Saad Z. A., Khodeer D. M., Zaitone S. A., Ahmed A. A. M., Moustafa Y. M. (2020). Life Sci..

[cit25] Zaitone S., Hassan N., El-Orabi N., El-Awady E.-S. (2011). Eur. J. Pharmacol..

[cit26] Pan M., Song Y. L., Xu J. M., Gan H. Z. (2006). J. Pineal Res..

[cit27] Allain C. C., Poon L. S., Chan C. S., Richmond W., Fu P. C. (1974). Clin. Chem..

[cit28] Fossati P., Prencipe L. (1982). Clin. Chem..

[cit29] Matthews D. R., Hosker J. P., Rudenski A. S., Naylor B. A., Treacher D. F., Turner R. C. (1985). Diabetologia.

[cit30] Qian M., Nahoum V., Bonicel J., Bischoff H., Henrissat B., Payan F. (2001). Biochemistry.

[cit31] Nafie M. S., Tantawy M. A., Elmgeed G. A. (2019). Steroids.

[cit32] Banerjee S. (2012). J. Assoc. Physicians India.

[cit33] Rosenstock J., Kim S. W., Baron M. A., Camisasca R.-P., Cressier F., Couturier A., Dejager S. (2007). Diabetes, Obes. Metab..

[cit34] Russell-Jones D., Cuddihy R. M., Hanefeld M., Kumar A., González J. G., Chan M., Wolka A. M., Boardman M. K. (2012). Diabetes Care.

[cit35] Kodama N., Tahara N., Tahara A., Honda A., Nitta Y., Mizoguchi M., Kaida H., Ishibashi M., Abe T., Ikeda H., Narula J., Fukumoto Y., Yamagishi S., Imaizumi T. (2013). J. Clin. Endocrinol. Metab..

[cit36] Shah P. K., Mudaliar S., Chang A. R., Aroda V., Andre M., Burke P., Henry R. R. (2011). Diabetes, Obes. Metab..

[cit37] McGuire D. K., Inzucchi S. E. (2008). Circulation.

[cit38] Derksen G. C. H., van.Beek T. A. (2002). Stud. Nat. Prod. Chem..

[cit39] Cuoco G., Mathe C., Archier P., Chemat F., Vieillescazes C. (2009). Ultrason. Sonochem..

[cit40] Ford L., Rayners C. M., Blackburn R. S. (2015). Phytochemistry.

[cit41] Essaidi I., Snoussi A., Ben Haj Koubaier H., Casabianca H., Bouzouita N. (2017). Pigm. Resin Technol..

[cit42] Martins D., Nunez C. V. (2015). Molecules.

[cit43] Proença C., Freitas M., Ribeiro D., Tomé S. M., Oliveira E. F. T., Viegas M. F., Araújo A. N., Ramos M. J., Silva A. M. S., Fernandes P. A., Fernandes E. (2019). J. Enzyme Inhib. Med. Chem..

[cit44] Rathee P., Chaudhary H., Rathee S., Rathee D., Kumar V., Kohli K. (2009). Inflammation Allergy: Drug Targets.

[cit45] Pietta P. G. (2000). J. Nat. Prod..

[cit46] Gow-Chin Y., Pin -Der D., Da-Yon C. (2000). Food Chem..

[cit47] Evans J. L., Goldfine I. D., Maddux B. A., Grodsky G. M. (2003). Diabetes.

[cit48] Gothai S., Ganesan P., Park S.-Y., Fakurazi S., Choi D.-K., Arulselvan P. (2016). Nutrients.

