# Correction: *Rubia tinctorum* root extracts: chemical profile and management of type II diabetes mellitus

**DOI:** 10.1039/d0ra90088e

**Published:** 2020-08-24

**Authors:** Enas E. Eltamany, Mohamed S. Nafie, Dina M. Khodeer, Aya H. H. El-Tanahy, Maged S. Abdel-Kader, Jihan M. Badr, Reda F. A. Abdelhameed

**Affiliations:** Department of Pharmacognosy, Faculty of Pharmacy, Suez Canal University Ismailia 41522 Egypt; Department of Chemistry, Faculty of Science, Suez Canal University Ismailia 41522 Egypt; Department of Pharmacology & Toxicology, Faculty of Pharmacy, Suez Canal University Ismailia 41522 Egypt; Damietta Health Affairs Directorate Damietta Egypt; Department of Pharmacognosy, College of Pharmacy, Prince Sattam Bin Abdulaziz University 173 AlKharj 11942 Saudi Arabia mpharm101@hotmail.com +966545539145; Department of Pharmacognosy, Faculty of Pharmacy, Alexandria University Alexandria 21215 Egypt

## Abstract

Correction for ‘*Rubia tinctorum* root extracts: chemical profile and management of type II diabetes mellitus’ by Enas E. Eltamany *et al.*, *RSC Adv.*, 2020, **10**, 24159–24168, DOI: 10.1039/D0RA03442H.

The authors regret that there was an error in the caption for Table 6 in the original article. The text originally read: “Flavonoids of *Rubia tinctorum* root extract identified by LC-HRMS analysis (positive mode)”. This caption should read: “Flavonoids of *Rubia tinctorum* root extract identified by LC-HRMS analysis (negative mode)”.

In addition, there was an error in one of the columns of Table 6 in the original article. The column was originally labelled “*m*/*z* [M + H]^+^”. This should read “*m*/*z* [M − H]^−^”.

The Royal Society of Chemistry apologises for these errors and any consequent inconvenience to authors and readers.

## Supplementary Material

